# Towards personalized recommendation with enhancing preference matching through scene-weighted reranking

**DOI:** 10.1371/journal.pone.0333097

**Published:** 2025-11-18

**Authors:** Kun Tong, GuoXin Tan

**Affiliations:** 1 College of Information Engineering, Hubei Polytechnic Institute, XiaoGan, People's Republic of China; 2 National Research Center of Cultural Industries, Central China Normal University, WuHan, People's Republic of China; The Catholic University of Korea, KOREA, REPUBLIC OF

## Abstract

Reranking is crucial in recommendation systems, refining candidate lists to significantly enhance the matching of user preferences and encourage engagement. While existing algorithms often focus solely on pairwise item interactions, they overlook local connections within item subsets. To address this limitation, we introduce the concept of “scenes” to explicitly mine local relationships among multiple items within a list, representing inter-scene correlations through undirected graphs. To effectively integrate these scenes and address the challenge of scoring items that cannot be definitively categorized into a single scene, we propose a scene-weighted reranking algorithm. This novel approach computes a final item score by leveraging scene-user preference matching scores, weighted by item-scene similarities. Experimental results demonstrate that compared to existing methods, our algorithm achieves more accurate item rankings that better reflect users’ true preferences, ultimately providing higher-quality recommendation sequences. This research contributes to the field by offering a more nuanced approach to capturing both local and global item relationships, specifically enhancing preference matching in personalized recommendation.

## 1. Introduction

Contemporary personalized recommendation systems typically encompass two stages: retrieval and personalized reranking, with the latter being crucial as it directly generates the results presented to users [1] Users expect these final recommendations to align with their interests and preferences, reflecting their user profiles. Thus, the reranking phase serves to reorganize initial retrieval results based on individual user inclinations. The reranking algorithm constructs a model considering both the relevance between user profiles and each item in the candidate list, as well as inter-item contextual relationships [2,3,4]. It optimizes a loss function incorporating pairwise influences between sample points and list-wise influences among sample clusters in the feature space, aiming to produce an optimal output sequence matching user preferences. Notably, as the original candidate list may contain items misaligned with user preferences, the optimal output sequence is typically shorter than the initial list [1,4].

Employing generative models as a foundational framework for reranking tasks presents a viable approach [5]. This methodology bifurcates the reranking process into two distinct phases: sequence generation and sequence evaluation. In the initial phase, the generative model produces a vast array of enumerated sequences based on the original candidate ranking, forming an enumeration set. Subsequently, in the evaluation phase, a pre-designed evaluator assesses each enumerated sequence, with the highest-scoring sequence selected as the optimal output for user presentation. This dual-phase framework is termed the evaluator-generator paradigm [6]. Within this paradigm, the generator’s capacity to produce diverse enumeration sets is paramount. Traditional generative models, such as GANs [7] and VAEs [8], have demonstrated limitations in this regard, impeding their efficacy in reranking tasks and hindering their application in recommendation models. Recently, diffusion models have achieved remarkable success across various computer vision domains. These models iteratively introduce noise into original samples during forward diffusion, subsequently learning to denoise and generate samples approximating the original distribution during reverse diffusion. Distinctively, diffusion models utilize Markov chains to learn noise distributions and generate data through denoising sampled data, theoretically offering significantly higher model capacity than traditional generative models [9]. Consequently, researchers have pioneered the integration of diffusion models into reranking algorithms with promising results [9]. However, the evaluator-generator paradigm necessitates the generation of sequences for evaluation in its initial phase, potentially incurring substantial computational costs when dealing with complex sequence items, such as high-resolution image data. This computational burden may significantly impact the overall efficiency of the reranking model, potentially limiting the practical applicability of the recommendation system [10].

To address the aforementioned issues, some researchers have proposed a scene-based reranking algorithm framework [11]. A scene refers to a local cluster of items in the feature space with semantically similar characteristics. For instance, when a female user is browsing for women’s jackets, a user-friendly recommendation sequence would position items like women’s dresses in close proximity to the jacket, while products such as over-ear headphones, which lack the “women’s clothing” semantic, would be placed further away. In this context, women’s jackets, dresses, and similar items form a scene in the feature space, with the local semantic representation of “clothing suitable for women.”

Generally, it is assumed that when users show interest in a specific item, such as an intention to purchase or click, they are likely to have similar intentions towards related or similar items within the same scene [12]. This implies that users tend to exhibit consistent preferences across all items within a particular scene. Researchers posit that the reranking task can be transformed into a process of identifying highly correlated scenes within the list using certain methods [13]. The semantic description of a scene can be represented by the feature vector of its key item. Subsequently, the algorithm calculates the degree of alignment between the user profile and each scene, then arranges the scenes in the list based on this alignment. The resulting sequence is then presented to the user, completing the reranking task. Notably, this approach does not compute the preference similarity between each item and the user profile. Instead, it calculates the similarity between key items in each scene and the user profile, significantly reducing computational costs and enhancing operational efficiency.

The construction of item groups within scenes is a crucial research topic, as models require representation of key items to represent entire scenes in computations. Some reseachers [1] proposed a clustering-like method to obtain scene representation vectors: first, constructing an inter-item relationship factor matrix through contrastive learning, then using the MDCA algorithm to partition the item set into multiple clusters, and finally using the average of items in the feature space within each cluster as the representation vector. Some reseachers [14] proposed using graph models, representing scenes as weighted undirected graphs, with nodes representing items and edge weights indicating items similarities. Graph neural networks are then applied to integrate scene structure and node information, yielding scene representation vectors [15].

After obtaining scene representations, the reranking process should be user profile- oriented. Existing research typically merges user profile vectors with scene vectors and inputs them into multilayer perceptrons (MLPs) to generate results [16,17] However, MLP weights heavily depend on the input order of scene vectors, with minor perturbations potentially causing significant differences. More importantly, real-world scenarios often contain items that are difficult to categorize into specific scenes, such as items in travel recommendations that simultaneously include natural and cultural attractions. This phenomenon described is commonly referred to as scene ambiguity [18]. Traditional scene-based reranking models merely consider the similarity relationship between items and their affiliated scenes, failing to explore the association strength of items with other scenes. In such cases, when an item exhibits scene ambiguity (i.e., where an item demonstrates high similarity with multiple scenes), this can significantly disrupt reranking results, thereby compromising the overall effectiveness of the model.

To address these issues, we propose a scene-weighted reranking approach to enhance preference matching. The model processes user profiles and items through separate transformer modules to generate contextual tokens, strengthening the learning of semantic correspondences between sequences and user profiles while improving training stability and robustness. Subsequently, we construct an inter-item preference similarity function to derive scene distributions from the candidate list and compute scene-user preference matching scores via dot product similarity. For each candidate item, the model calculates preference similarities with multiple scenes, performs polynomial multiplication with the scene-user matching scores to obtain weighted sums, and maps these aggregates to final scores through a mapping function. The reranking mechanism then optimizes the output sequence based on these scores.

The introduction of scene-weighted sums allows the model to perform mixed calculations between items and global scenes, rather than being limited to single-scene ranking. This effectively mitigates the efficiency degradation caused by scene ambiguity, enhancing the model’s generalization ability, adaptability and preference matching in personalized recommendation.

## 2. Related works

### Item-level Reranking

This method reorders items based on their mutual influences. [19] and [20] proposed using RNN networks to encode item-level sequences into high-dimensional feature spaces and calculate influence factors between feature vectors. However, these methods are constrained by network architecture limitations and high computational costs for long sequence encoding. [21] introduced a self-attention mechanism to compute inter-item correlations for reranking. Similarly, [14] employed graph models to calculate item correlations, yielding favorable results. [22] further advanced this concept by utilizing Transformer modules to capture inter-item relationships. [23] addressed the information loss problem in long sequence reranking by proposing a feature augment solution.

### List-level Reranking

In contrast to item-level methods, list-level methods [19,4,10,24] treat the entire list as a unit, evaluating scores between different lists and selecting the highest-scoring list order as the final reranking result. List-level reranking is generally categorized into two types:The first type [25,26,27] models the overall distribution relationships among list items and employs learnable scoring functions to evaluate lists.The second type [28,29,30] views reranking as a two-stage process: sequence generation and sequence scoring. It uses a generator to produce various sequences and incorporates an evaluation module at the model backend to assess each generated sequence, ultimately selecting the optimal sequence as the final model output.

### Scene-based Reranking

The scene-based reranking algorithm was initially proposed in [31]. This research aims to address the challenge of mining local similarity distributions of semantic features among elements within recommendation lists. By pre-partitioning the recommendation lists, the approach enhances the performance and efficiency of recommendation algorithms.Building upon this algorithm, [11] employs multi-head attention to model interactions between scenes and derives high-quality embeddings of users and items through a mask pretraining mechanism, thereby achieving more stable recommendation system performance.

## 3. Methods

This chapter is organized as follows: Conceptualization of Scene section provides a formal conceptualization of the Scene. Acquisition of User Representations and Item Embeddings **s**ection elucidates the pre-training methodology for deriving user representations and item embeddings. Extraction of Scene feature representations section presents a schematic representation of the proposed model and delineates the process of extracting Scene feature representations. Extraction of Scene Embeddings section explicates the utilization of a multi-head attention mechanism for generating Scene embeddings. Items Reranking section articulates the computational framework for final user embeddings and item scores, which serve as the basis for reranking the candidate list to yield an optimal output sequence. The chapter concludes with Optimization section, which specifies the loss function employed during the model’s training phase.

### 3.1. Conceptualization of scene

This section elucidates the concept of “Scene” as employed in our research framework. We define a Scene as a collection of items forming a localized adjacency structure within the feature space, characterized by high local relative influence factors among its constituents. To operationalize and leverage these factors, we establish local relationship metrics, primarily focusing on similarity and diversity. Each Scene is anchored by a key item that encapsulates its overall characteristics, while the constituent items maintain a delicate balance of high intra-Scene similarity and inter-item distinctiveness. This formulation adheres to the dual criteria of similarity and diversity. By restructuring the original candidate list into multiple Scene-based arrangements, we facilitate a more nuanced and comprehensive analysis of inter-item associations, thereby enhancing the model’s capacity for contextual understanding and personalized reranking. Quantifying inter-item local relative influence factors requires a judicious selection of a metric function $pr(v_{i},v_{j})$,Wherein $v_{i}$ and $v_{j}$ represent arbitrary, non-identical items from the candidate set. The function $pr(v_{i},v_{j})$ can represent any arbitrary similarity metric capable of measuring the resemblance between vectors $v_{i}\;$ and $v_{j}$, such as cosine similarity or other distance-based measures. Consider a Scene $S=\{v_{s\text{1}},v_{s\text{2}},\cdots v_{sk}\}$ encompassing a pivotal item $v_{o}\in S$. To ensure that the constituents of S concurrently manifest both similarity and diversity in their local relationships, the following constraints must be satisfied:


$$c_{i}\neq c_{j}\;,\;\;\forall v_{i},v_{j}\in S$$ (1)

$$pr(v_{i},v_{o})>\gamma \;,\;\forall v_{i}\in S$$ (2)

Herein, $c_{i}$ and $c_{j}$ denote the categorical classifications of $v_{i}\;$ and $v_{j}$, respectively, corresponding to their assigned labels. γ represents a threshold constant, implemented to ensure a cohesive preference relationship among all constituents within a given scene. This parameter additionally regulates scene granularity, thereby ultimately governing diversity within the final recommendation sequence.Observable from the aforementioned constraints, condition (1) mandates that all items within a single Scene belong to distinct categories, thus preserving diversity in local relationships. Condition (2) stipulates that the similarity metric between any arbitrary pair of items must surpass a predetermined threshold, thereby maintaining the local relationship of similarity. Given a predefined upper bound n on Scene cardinality, the initial candidate set $\left\{v_{\text{1}},v_{\text{2}},\cdots ,v_{q}\right\}$ can be decomposed into a sequence of Scenes $\left\{S_{\text{1}},S_{\text{2}},\cdots ,S_{p}\right\}\;\;,p\leq n$ where each Scene adheres to the aforementioned constraints.

### 3.2. Acquisition of user representations and item embeddings

**Fig 1:**
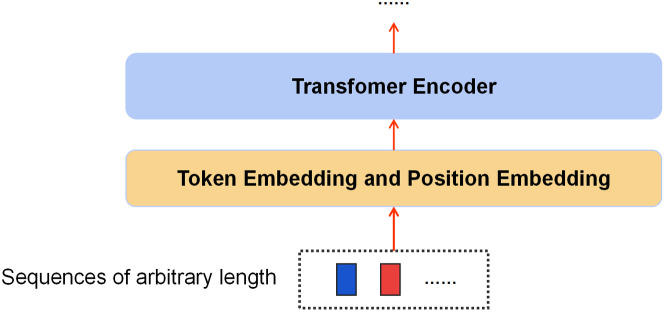
Conceptual schematic of the architecture for pre-training item and user representational embeddings.

The acquisition of precise user representations and initial item embeddings is paramount for optimizing the efficiency of reranking models. Conventional direct initialization techniques frequently result in performance instability and diminished robustness. To mitigate these issues, we propose a pre-training methodology utilizing a Transformer encoder,drawing inspiration from advancements in Computer Vision (CV) and Natural Language Processing (NLP). This approach, demonstrating superiority over traditional architectures such as recurrent neural networks, efficiently processes variable-length sequences, thereby capturing global semantic information for both users and items. The Transformer’s inherent capability to discern contextual relationships in arbitrary-length sequences ensures the generation of robust embeddings that encapsulate holistic semantic knowledge. Consequently, this methodology significantly enhances the overall performance and stability of the reranking process. The architectural framework of this pre-training model is delineated in Fig 1, providing a visual representation of our proposed approach.

Given a set of initial candidate sequences $u=\{v_{\text{1}},v_{\text{2}},\cdots ,v_{k}\}$, a distinctive token [CLS] is affixed to the anterior of each sequence, serving as a categorical identifier for the entire sequence.For an arbitrary element $v_{i}\in u$, a transformation is required to convert its item feature vectors into corresponding embedding $p_{i}$:


$$p_{i}=Token(v_{i})$$ (3)

In this context, the function Token(·) denotes the token embedding layer within the transformer architecture. Consequently, the candidate sequence u can be represented as a set of embeddings $P=\{p^{cls},p_{\text{1}},p_{\text{2}},\cdots ,p_{k}\}$. These embedding are then propagated through the Transformer encoder layers to derive the final embedding for each constituent item:


$$P^{\prime }\;=\;Transformer(P)$$ (4)

Leveraging the delineated methodology, we derive the definitive item embedding vectors. With respect to user embeddings, exploiting the transformer’s capability to process sequences of arbitrary length, we conceptualize the user as a singular instance of the original candidate sequence $user=\{{user}_{\text{1}}\}$, where |user|=1. Applying the aforementioned algorithmic process to this construct yields the initial user embedding ${user}^{\prime }$ and the final item embedding $P^{\prime }=\{q_{\text{1}},q_{\text{2}},\cdots ,q_{n}\}$.

### 3.3. Extraction of scene feature representations

**Fig 2:**
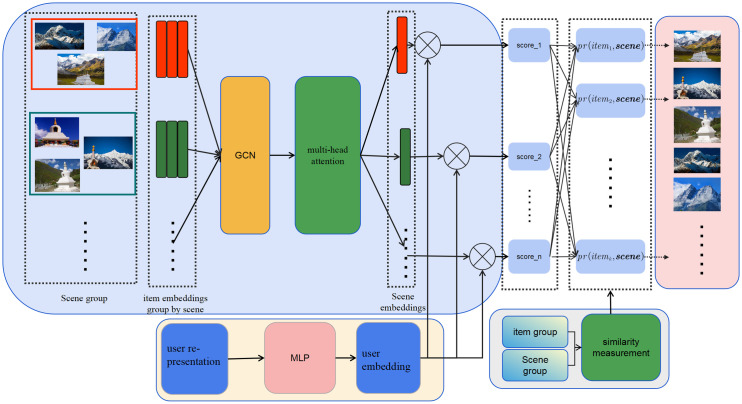
Architectural diagram delineating the comprehensive structure of our proposed model.


Fig 2 provides a schematic representation of the holistic architectural framework of our proposed model.Upon acquiring the embedding vectors for all items within the initial candidate list, the model’s principal objective is to perform scene-based segmentation of the candidate list and derive the corresponding scene embeddings. Given a sequence of scene features Scene that adheres to constraints (1) and (2), we utilize a Graph Convolutional Network (GCN) [11] to learn the feature representation for each scene.GCN are specifically designed to process graph-structured input data, it is imperative to transform the original list structure of our sequence into an equivalent graph representation to facilitate the application of GCN to our task. First, following the formal definition of scenes established in Conceptualization of Scene section, the entire item set undergoes scene partitioning to generate a structured sequence of scenes $S_{\text{1}},S_{\text{2}},\cdots S_{k}$. For an arbitrary Scene $S_{e}$, in accordance with the mathematical formulation proposed in Conceptualization of Scene section, we construct its corresponding graph structure G using the following formalism:

$$\begin{array}{c} G_{i,j}=\left\{ \begin{array}{c} @lpr(v_{i},v_{j}),\;\;pr(v_{i},v_{j})>\gamma \;and\;i\neq j\\ \text{0},\;\;otherwise \end{array}\right.\; \end{array}$$ (5)

The parameter γ herein is congruent with γ in constraint (2). This methodology guarantees that exclusively highly correlated items are incorporated into an identical graph structure G. Moreover, given that scenes must preserve inter-item similarity and be characterized by key item representations to delineate the scene’s distribution in feature space, our approach mandates the construction of an adjacency matrix. This matrix serves to quantify the influence between adjacent nodes within the graph structure:


A=I+αNorm(G) (6)

Where I denotes the identity matrix, and Norm(·) represents a normalization function that applies L1 regularization to each row vector of the input matrix.Subsequently, given a scene $S_{e}=\{v_{s\text{1}},v_{s\text{2}},\cdots v_{sk}\}$ containing a key item $v_{o}$, we can derive the scene’s representation s according to the following formula:


$$s=\;A_{o}P_{S_{e}}W$$ (7)

Where $A_{o}\in R^{\text{1}\times k}$ denotes the column vector in the adjacency matrix A corresponding to the key item $v_{o}$, $P_{S_{e}}\in R^{k\times d}$ represents the concatenation matrix of embedding vectors for all items in scene $S_{e}$, with item embedding having been obtained during the pre-training phase. d denotes the dimension of the embedding, and W represents a learnable parameter matrix. By applying this method to process the candidate list sequence, we can automatically learn the representation for each scene during the training phase.

### 3.4. Extraction of scene embeddings

To mitigate the challenge of output volatility stemming from varied input sequences of scene representation, we implement a multi-head attention mechanism. This approach facilitates the learning of global inter-sample influence factors, consequently yielding more cohesive scene embeddings.Consider a scene representation matrix $S\in R^{k\times d_{s}}$, which comprises the concatenation of all scene representation $\left\{s_{\text{1}},s_{\text{2}},\cdots s_{k}\right\}$ from the initial candidate list, where $d_{s}\;\in N$ denotes the dimensionality of each scene representation. The attention values for this scene representation matrix can be computed using the following formulation:


$$Att\left(S\right)=softmax\left(\frac{SW_{Q}{\left(SW_{K}\right)}^{T}}{\sqrt{d_{s}}}\right)SW_{V}$$ (8)

Where $W_{Q}\in R^{d_{s}\times d_{k}}$, $W_{K}\in R^{d_{s}\times d_{k}}$ and $W_{V}\in R^{d_{s}\times d_{k}}$ represent learnable parameter matrices, which are integral to the generation of queries, keys, and values, respectively, within the transformer architecture. The function softmax:R→[0,1] serves to map the computational output to the unit interval. This methodology enables the automatic derivation of inter-scene influence factors, manifested as attention values.

We can now derive a novel scene embedding matrix $S_{M}\in R^{k\times d_{s}}$ via the multi-head attention mechanism, transforming the initial scene representation matrix and incorporating h distinct mutual influence factors:


$$S_{M}=\left[{Att\left(S\right)}_{\text{1}},{Att\left(S\right)}_{\text{2}},\cdots ,{Att\left(S\right)}_{n_{e}}\right]$$ (9)

$$where\;\;\;{Att\left(S\right)}_{i}=softmax\left(\frac{SW_{Q}^{i}{\left(SW_{K}^{i}\right)}^{T}}{\sqrt{d_{s}}}\right)SW_{V}^{i}\;\;\;\forall i\in \left[\text{1}:n_{e}\right]$$ (10)

Where [•, •] denotes the concatenation operator, representing the operation of con- catenating all elements within the parentheses along the row dimension. $n_{e}$ represents the number of input layer nodes in the multi-head attention mechanism. $\left\{W_{Q}^{i},W_{K}^{i},W_{V}^{i}\right\}$ signifies the learned parameter matrix in the i-th attention module. Finally, to enhance the quality of scene embedding representations, the computational results are processed through a feed-forward neural network after the attention module:


$$S_{E}=FFN\left(S_{M}\right)=\text{max}\left(\text{0},S_{M}W_{F}^{\text{1}}+b^{\text{1}}\right)W_{F}^{\text{2}}+b^{\text{2}}$$ (11)

Let $X\in \{W_{F}^{\text{1}},W_{F}^{\text{2}},b^{\text{1}},b^{\text{2}}\}$denote the set of learnable parameters employed in the feed-forward neural network, where $W_{F}^{\text{1}}\in R^{d_{s}\times d_{f}}$,$W_{F}^{\text{2}}\in R^{d_{f}\times d_{s}}$ are weight matrices and $b^{\text{1}}$, $b^{\text{2}}$ are bias vectors. $d_{f}\;\in N$ represents the dimensionality of the feed-forward network’s hidden layer. Through iterative application of multi-head attention modules and feed-forward networks, we derive high-fidelity scene embeddings $S_{E}^{n_{e}}$. These refined scene embeddings are then integrated with user representation embeddings to compute the terminal scores for individual items.

### 3.5. Items reranking

To maximize the congruence between the final ranking and user preferences, we compute a preference score for each item within the candidate list. Subsequently, these items are arranged in descending order of their computed scores, yielding a partially ordered sequence that is then presented to the user.For an arbitrary user $u^{i}$, we utilize a multilayer perceptron to transform the user’s initial representation user into a deep embedding vector ${user}_{e}$.


$${user}_{e}=\text{MLP}\left(user\right)=W_{U}user+b_{U}$$ (12)

Let $A=\{W_{U},b_{U}\}$ denote the set of learnable parameters of the multilayer perceptron, where $W_{U}$ and $b_{U}$ represent the weight matrix and bias vector for the i-th layer, respectively. This multilayer perceptron facilitates the mapping of the user’s initial embedding vector to a feature space that is isomorphic to that of the scene embedding vectors. Consequently, we can compute preference scores independently between the user features and any scene within the candidate list:


$$r={user}_{e}⊗S_{E}^{n_{e}}=\left\{{{user}_{e}}^{T}{\tilde{s}}_{\text{1}},{{user}_{e}}^{T}{\tilde{s}}_{\text{2}},\cdots ,{{user}_{e}}^{T}{\tilde{s}}_{k}\right\}$$ (13)

Where ⊗ denotes the vector inner product operation, and ${\tilde{s}}_{i}$ represents the i-th scene representation in the scene embedding $S_{E}^{n_{e}}$. Subsequently, for any item $v_{m}$ in the candidate list, we compute its similarity matching score with all scenes:


$$r_{v}=pr\left(v_{m},v_{i}^{o}\right)=\left\{pr\left(v_{m},v_{\text{1}}^{o}\right),pr\left(v_{m},v_{\text{2}}^{o}\right),\cdots ,pr\left(v_{m},v_{k}^{o}\right)\right\}$$ (14)

Let $v_{i}^{o}$ denote the key item in the i-th scene. We then derive the final score for item $v_{m}$ by calculating a polynomial weighted sum of the user-scene preference score r and the item-scene matching score $r_{v}$:


$$\begin{array}{c} r_{v_{m}}=r⊕r_{v}=\sum {\mathrm{\backslash nolimits}}_{i=\text{1}}^{k}pr\left(v_{m},v_{i}^{o}\right){{user}_{e}}^{T}{\tilde{s}}_{i} \end{array}$$ (15)

The unbounded nature of $r_{v_{m}}$’s range presents challenges for optimization using conventional loss functions. Consequently, rather than directly utilizing $r_{v_{m}}$ as the terminal score, we have implemented a refinement to augment its scalability:


$$\tilde{r_{v_{m}}}=clip\left(r_{v_{m}}\right)$$ (16)

Where clip(·) represents a function that maps the input parameters to the interval [0,1]. For all items in the original candidate list, we compute their corresponding final scores according to equation (16). Once we have obtained the final scores for all items, we can rank the original candidate list based on these scores, thereby deriving the optimal output sequence and completing the reranking of the original candidate list.

### 3.6. Optimization

The optimization objectives for reranking models primarily encompass two frameworks: list-wise and point-wise approaches. List-wise functions, predominantly employed in generative reranking models, evaluate the quality of stochastically generated reranking sequences to compute model loss. However, this method necessitates exhaustive enumeration of all possible permutations, presenting significant computational challenges. Conversely, point-wise functions directly assess individual items within the original candidate list, circumventing the need for comprehensive permutation evaluation. This approach substantially reduces computational overhead and enhances model efficiency. Given these considerations, this study adopts a point-wise optimization function as the model’s objective function, leveraging its computational efficacy and scalability in the context of our reranking task.

For a given user ${user}_{u}$, we employ the mean squared error loss function to quantify the discrepancy between the model’s predicted scores and the item label values. Consider an arbitrary item $v_{m}$ from the original candidate list, a given scene list $s=\{s_{\text{1}},s_{\text{2}},\cdots ,s_{k}\}$, and the item’s final score ${\tilde{r}}_{v_{m}}$. The item label value is computed as follows:


$$\begin{array}{c} {label}_{m}=\sum {\mathrm{\backslash nolimits}}_{i=\text{1}}^{k}pr\left(v_{m},v_{i}^{o}\right)r_{o}^{i} \end{array}$$ (17)

$$\tilde{{label}_{m}}=clip\left({label}_{m}\right)$$ (18)

Let $v_{i}^{o}$ denote the key item in the i-th scene, $r_{o}^{i}$ represent the final score of the key item in the i-th scene, and clip(·) be a function mapping input parameters to the unit interval. The model’s overall loss function is then defined as:


$$\begin{array}{c} Loss=\frac{\text{1}}{\text{2}}{\sum {\mathrm{\backslash nolimits}}_{i=\text{1}}^{q}\tilde{{(label}_{i}-}{\tilde{r}}_{v_{i}})}^{\text{2}} \end{array}$$ (19)

Let q denote the cardinality of the original candidate set, while $\tilde{{label}_{i}}$ and ${\tilde{r}}_{v_{i}}$represent the label value and final score for item $v_{i}$, computed using equations (18) and (16) respectively.

## 4. Experiments

### 4.1. Dataset and evalution metric

This study utilizes three public datasets: MovieLens-1M, MovieLens-20M [32], and the TripAdvisor dataset [33] for comparative experiments between our proposed model and other benchmark models. Following the methodology in [31], samples with user ratings exceeding 3 are designated as positive, while others are considered negative. For each dataset, we limit positive samples per user to approximately 15, subsequently ordering them chronologically by timestamp. The initial 80% of positive samples constitute the training set, with the remaining 20% forming the test set. During training, all positive samples are selected as key items, with $n_{s}-\text{1}$ highly correlated but non-positive samples randomly chosen to construct scenes. This rigorous data preparation ensures a balanced and temporally consistent evaluation framework, facilitating robust model comparison and performance assessment across diverse recommendation scenarios. To evaluate our proposed model performance, we employ four metrics for comparative analysis between our proposed model and benchmark models: NDCG@N, Precision@N, Recall@N, and SR@N. The first three metrics—NDCG@N, Precision@N, and Recall@N—are widely adopted for assessing the quality of output sequences in recommender systems, providing comprehensive insights into ranking accuracy and relevance. SR@N, in contrast, serves as a measure of local inter-item relationships within the list, offering a unique perspective on the model’s ability to capture item-item interactions. This diverse set of metrics ensures a multifaceted evaluation of model performance, encompassing both traditional recommendation quality indicators and more nuanced measures of item relationship modeling.

Following the definition proposed in [11], given a recommendation sequence $List=\{{item}_{\text{1}},{item}_{\text{2}},\cdots ,{item}_{N}\}$, we construct multiple scenes from it. Let ${item}_{i}$ be the key item, with i initially set to 1. We then find the maximum value K such that $\left\{{item}_{i},{item}_{i+\text{1}},\cdots ,{item}_{i+k}\right\}$ is a scene with ${item}_{i}$ as the key item, where K can be 0. Then, we set i to i + k + 1 and repeat the above steps until the last item ${item}_{N}$ in the recommendation sequence is included in the last scene. Let PS represent the number of scenes containing more than one item, and NS denote the number of scenes containing only one item. The SR@N is then defined as follows:


$$SR@N=\frac{PS}{PS+NS}$$ (20)

### 4.2. Implementation details

In accordance with the parameter optimization framework delineated in [11], we conduct an extensive hyperparameter search to determine the optimal configuration for our model. The resulting parameter settings, derived through rigorous experimentation and validation, are as follows:γ=0.6、α∈{0.001,0.01,0.1}、$d_{s}\in \{\text{1}\text{0},\text{2}\text{0},\text{3}\text{0},\text{4}\text{0},\text{5}\text{0}\}$、$d_{f}\in \{\text{1}\text{0},\text{2}\text{0},\text{3}\text{0},\text{4}\text{0},\text{5}\text{0}\}$、$n_{s}\in \{\text{1},\text{2},\text{3},\text{4},\text{5}\}$ and $n_{e}\in \{\text{1},\text{2},\text{4},\text{8}\}$. In our experimental framework, the parameter N is uniformly set to N={5,10,15}across all evaluation metrics. To ensure statistical robustness and representativeness, we compute the mean performance of the model on the test set for each metric.

### 4.3. Benchmark model

This section introduces the benchmark models used for comparison with our proposed model in the experiments. These benchmark models are widely employed in various recommender systems’ reranking modules:

- PRM [21]: This model utilizes a Transformer module to capture global semantic relationships between any pair of items in the list. It encodes related items into embedding vectors containing these semantic relationships. Subsequently, it concatenates user and item embeddings, feeding them into a neural network to obtain final scores. The items in the list are then ranked based on these scores to form the optimal output sequence.
- SRR [31]: This model pioneered the application of the scene concept in reranking. It divides the candidate list into multiple scenes using a clustering-like method. It then obtains feature representations for each scene through a graph neural network. These representations are fed into a multi-head attention module to compute embedding vectors for each scene. The model then calculates the matching degree between each scene embedding vector and the user, deriving scores for each scene. Finally, all scenes are ranked according to their scores, with items within each scene undergoing a secondary ranking based on their order of inclusion, resulting in the optimal output sequence.
- SRR-MP [11]: This model’s primary operational process is identical to SRR. However, before dividing the candidate list into scenes, it obtains embedding vectors for all items in the list through masked pre-training. It then divides scenes based on item embedding vectors rather than item feature representations. Compared to SRR, SRR with MR demonstrates more robust performance and stable operation across various recommendation tasks.
- PEAR [22]: This model divides the reranking task into two parts: 1) Learning intrinsic feature-level relationships between users and items. 2) Learning intrinsic instance- level relationships between items, considering user historical behavior as a reference. In the feature-level intrinsic relationship learning, user profile representations, initial list item representations, and user historical behavior items are directly merged and fed into a feature association module to mine intrinsic connections between list items, forming a set of vectors containing contextual semantic representations of the list. In the instance-level learning part, the model uses user historical behavior as guidance for the reranking process, employing multi-layer self-attention modules and cross- attention merge modules to calculate each item’s potential attractiveness to the user, finally ranking based on attractiveness.
- DCDR [9]: This model employs a generator-evaluator paradigm, applying diffusion models to reranking tasks. The overall model consists of two parts: 1) Forward separation process, 2) Backward reference process. In the forward separation process, noise is added to all samples in the initial sequence through a separation operator with a processable backend. In the backward reference process, a denoising model learns the original sequence’s feature space to generate relevant candidate sequences. Finally, a backward feedback mechanism is introduced to search for the highest-scoring sequence to recommend to the user.
- PODM-MI [34]: This model simultaneously learns from the initial item sequence and user profile. For the user profile processing branch, the model uses a variational inference-based multidimensional Gaussian distribution to capture users’ diverse uncertain preferences. In the sequence processing branch, it employs a backbone network to obtain users’ deterministic preferences and item feature embeddings. Finally, the model maximizes the mutual information between users’ diverse preferences and deterministic preferences, constructing a relationship matrix to guide item reranking.

### 4.4. Comparisions with state-of-the-art

The evaluation results of our proposed model in comparison with other benchmark models on the MovieLens-1M, MovieLens-20M, and TripAdvisor datasets are presented in Tables 1, 2, and 3, respectively. These results consistently demonstrate the superior performance of our proposed method in reranking tasks across all datasets.

**Table 1:** Performance evaluation using the MovieLens-1M benchmark dataset.

Method	NDCG@5	NDCG@10	NDCG@15	P@5	P@10	P@15	R@5	R@10	R@15	SR@5	SR@10	SR@15
PRM	0.1472	0.1458	0.1395	0.0395	0.0731	0.1021	0.1324	0.1378	0.1445	0.0337	0.0341	0.0336
SRR	0.1723	0.1649	0.1546	0.0446	0.0822	0.1165	0.1567	0.1709	0.1779	0.4867	0.5534	0.5515
SRR-MP	0.1812	0.1714	0.1621	0.0502	0.0967	0.1248	0.1732	0.1921	0.2009	0.5076	0.5598	0.5733
PEAR	0.1654	0.1602	0.1588	0.0501	0.0876	0.1163	0.1645	0.1756	0.1855	0.0597	0.0613	0.644
DCDR	0.1879	0.1822	0.1793	0.0589	0.1012	0.1297	0.1819	0.2045	0.2256	0.0531	0.0594	0.0609
PODM-MI	0.1923	0.1887	0.1841	0.0602	0.1021	0.1273	0.1834	0.2053	0.2271	0.0527	0.0548	0.0554
**Ours**	0.1987	0.1913	0.1877	0.0647	0.1101	0.1316	0.1889	0.2115	0.2306	0.5312	0.5713	0.5885

**Table 2:** Performance evaluation using the MovieLens-20M benchmark dataset.

Method	NDCG@5	NDCG@10	NDCG@15	P@5	P@10	P@15	R@5	R@10	R@15	SR@5	SR@10	SR@15
PRM	0.1673	0.1602	0.1524	0.0465	0.0769	0.0978	0.1261	0.143	0.1557	0.0321	0.0339	0.0345
SRR	0.2147	0.2011	0.1944	0.0628	0.836	0.1009	0.2255	0.2367	0.2494	0.4742	0.4813	0.5058
SRR-MP	0.2319	0.227	0.2201	0.0744	0.0979	0.1353	0.2351	0.2472	0.2583	0.5019	0.5158	0.5243
PEAR	0.2197	0.2125	0.2058	0.0715	0.1036	0.1283	0.229	0.2403	0.2547	0.0723	0.0788	0.0824
DCDR	0.253	0.2442	0.2356	0.0809	0.1058	0.1482	0.2526	0.2688	0.281	0.0707	0.0753	0.0851
PODM-MI	0.2628	0.2578	0.2512	0.0868	0.1221	0.174	0.2635	0.2719	0.2878	0.0756	0.0798	0.0854
**Ours**	0.2731	0.2657	0.2588	0.0916	0.1365	0.1801	0.2731	0.2889	0.2993	0.5155	0.5301	0.5417

**Table 3:** Performance evaluation using the TripAdvisor benchmark dataset.

Method	NDCG@5	NDCG@10	NDCG@15	P@5	P@10	P@15	R@5	R@10	R@15	SR@5	SR@10	SR@15
PRM	0.2488	0.2397	0.2253	0.0339	0.0607	0.0695	0.2015	0.2117	0.2258	0.0483	0.0491	0.0501
SRR	0.2695	0.2414	0.2358	0.0387	0.0698	0.0834	0.2607	0.2785	0.2902	0.5769	0.5521	0.5414
SRR-MP	0.2852	0.276	0.2631	0.0558	0.0834	0.0941	0.2913	0.2982	0.3047	0.604	0.5862	0.5716
PEAR	0.2712	0.248	0.2397	0.0419	0.0735	0.0907	0.2685	0.2844	0.2973	0.0492	0.0498	0.0504
DCDR	0.3148	0.3002	0.2911	0.0536	0.0887	0.1023	0.3001	0.3124	0.322	0.0523	0.0565	0.0596
PODM-MI	0.3175	0.3066	0.2989	0.0547	0.0903	0.1059	0.2989	0.3135	0.3267	0.0518	0.0546	0.0577
**Ours**	0.3202	0.3117	0.3036	0.0589	0.0936	0.1088	0.3045	0.3196	0.3303	0.621	0.6013	0.5897

In the MovieLens-1M datasets experiments, our model achieves an NDCG@15 of 0.1877, outperforming the best benchmark model, PODM-MI, by 1.9%. The P@15 metric reaches 0.1316, a 3.2% improvement over PODM-MI, while R@15 attains 0.2306, representing a 1.5% increase compared to PODM-MI.

For the MovieLens-20M datasets, PODM-MI again emerges as the top-performing benchmark. Our model surpasses PODM-MI by 3.02%, 3.5%, and 4% in NDCG@15, P@15, and R@15 metrics, respectively, demonstrating consistent improvement across all evaluation criteria.

In the TripAdvisor datasets experiments, PODM-MI maintains its position as the best-performing benchmark. Our proposed model exhibits improvements of 1.5% in NDCG@15, 2.6% in P@15, and 1.1% in R@15 compared to PODM-MI, further validating its effectiveness across diverse datasets.

Regarding the SR@N metric, PRM, PEAR, DCDR, and PODM-MI models show relatively poor performance across all three datasets. The highest SR@15 value among these models is achieved by PODM-MI on MovieLens-20M, reaching only 0.0854. In contrast, SRR, SRR-MP, and our proposed model demonstrate robust performance across all datasets, with most values falling within the [0.5, 0.6] range. Specifically for the SR@15 metric, SRR-MP emerges as the best-performing benchmark model. Our model surpasses SRR-MP by 2.65%, 3.31%, and 3.17% on the three datasets respectively, indicating substantial improvements in capturing local item relationships.

- According to the definition of the SR@N metric, a higher number of scenes with item cardinality greater than one results in a higher SR@N value. The poor performance of PRM, PEAR, DCDR, and PODM-MI models on this metric can be attributed to their approach in processing item interactions and relationships.The superior performance of SRR, SRR-ML, and our proposed method on the SR@N metric can be attributed to their focus on sample point clustering phenomena in the feature space during the embedding stage. These approaches delineate scenes comprising multiple highly correlated items, deriving embedding vectors for subsequent processing. In contrast, PRM, PEAR, DCDR, and PODM-MI, despite capturing inter-item interactions, primarily process individual items during feature embedding, overlooking localized clustering structures. This results in scenes predominantly composed of single items and a more dispersed distribution. The ability of SRR, SRR-ML, and our method to explicitly model localized structures leads to a higher number of scenes with item cardinality exceeding one, thus outperforming the other models on the SR@N metric. This observation underscores the importance of incorporating spatial awareness and cluster-based processing in recommendation systems, particularly for tasks requiring a nuanced understanding of intricate item relationships.
- Comparative analysis reveals our proposed method’s most significant improvement over the PRM model across all metrics. This enhancement is primarily attributed to our comprehensive modeling approach. While PRM exclusively focuses on global semantic relationships within the list, neglecting localized inter-item influences, our method incorporates both global semantics and local item correlations through the concept of scenes. By integrating this dual-perspective approach, our model demonstrates a more nuanced understanding of item relationships, leading to more effective reranking decisions. The incorporation of scene-based modeling enables the capture of subtle, localized patterns often overlooked in purely global approaches. This marked superiority underscores the importance of considering multi-scale relationships in recommendation systems, suggesting that future advancements in reranking algorithms could benefit from balancing global semantic understanding with granular item relationships for more accurate and context-aware recommendations.
- Among PEAR, DCDR, and PODM-MI models, PEAR exhibits the largest performance gap compared to our proposed method, while still significantly outperforming the PRM model. This performance hierarchy can be attributed to the varying approaches in modeling local item relationships. PEAR explicitly models local item interactions but overlooks the localized clustering structures (scenes) in the feature space, potentially leading to suboptimal item placements in the final reranking. In contrast, DCDR and PODM-MI, while also not explicitly focusing on localized structures, compensate for this limitation through distinct methodologies. PODM-MI employs variational inference to adaptively optimize each item’s optimal recommendation position, while DCDR leverages a noise-addition and denoising process to directly learn user-preference-aligned item feature distributions, guiding subsequent reranking. Our method’s superior performance underscores the importance of comprehensively modeling both global semantics and localized item clusters, suggesting that future advancements in reranking algorithms could benefit from integrating these multi-faceted approaches to capture complex item relationships more effectively.
- Among models that incorporate scene, this model demonstrates superior performance. While SRR and SRR-ML achieve noteworthy results by modeling the local clustering structure of items, they only consider item-scene similarity in the final scoring phase, neglecting relevance to other scenes. Moreover, their ranking approach, which directly determines item position based on scene ranking, leads to suboptimal placement of items with scene ambiguity but high user preference, resulting in overall efficiency decline. In contrast, this model addresses items with scene ambiguity by calculating item-scene relevance across all scenes and employing a weighted sum with scene-user matching scores. This approach enables the model to consider item ranking from a global perspective encompassing all scenes present in the list, rather than limiting focus to an item’s primary scene. Consequently, items with scene ambiguity are positioned more accurately within the ranking, enhancing the model’s overall precision.

### 4.5. Ablation experiments

This section evaluates the impact of the newly proposed components – the feature embedding layer and the item-scene similarity function – on model performance. Specifically, the following variant models are constructed:

- W/O FE: The feature embedding layer proposed in Acquisition of User Representations and Item Embeddings section is removed from the original model and replaced with an identity mapping.
- W/O SSE: The weighted scene scoring function introduced in Items Reranking section is eliminated, with all similarity matching scores set to a uniform weight of 1.

The experimental parameters align with those described in Implementation details section. Ablation studies are conducted on the MovieLens-1M, MovieLens-20M, and TripAdvisor datasets. Evaluation metrics include NDCG@N, Precision@N, Recall@N, and SR@N(where SR@N follows the definition in Dataset and Evalution Metric section, with N set to *N* = {5, 10, 15}).Results are presented in Tables 4–6.

**Table 4:** Comparative results of ablation experiments on the MovieLens-1M dataset.

Method	NDCG@5	NDCG@10	NDCG@15	P@5	P@10	P@15	R@5	R@10	R@15	SR@5	SR@10	SR@15
W/O FE	0.1755	0.1712	0.1698	0.0537	0.0976	0.1143	0.1781	0.1965	0.2072	0.5089	0.5325	0.5411
W/O SSE	0.1708	0.1684	0.1646	0.0524	0.0957	0.1056	0.1740	0.1907	0.2005	0.4873	0.5071	0.5197
**Ours**	0.1987	0.1913	0.1877	0.0647	0.1101	0.1316	0.1889	0.2115	0.2306	0.5312	0.5713	0.5885

**Table 5:** Comparative results of ablation experiments on the MovieLens-20M dataset.

Method	NDCG@5	NDCG@10	NDCG@15	P@5	P@10	P@15	R@5	R@10	R@15	SR@5	SR@10	SR@15
W/O FE	0.2518	0.2479	0.2337	0.0837	0.1246	0.1664	0.2613	0.2699	0.2756	0.5053	0.5125	0.5249
W/O SSE	0.2462	0.2435	0.2281	0.0798	0.1203	0.1576	0.2577	0.2608	0.2685	0.4875	0.5022	0.5118
**Ours**	0.2731	0.2657	0.2588	0.0916	0.1365	0.1801	0.2731	0.2889	0.2993	0.5155	0.5301	0.5417

**Table 6:** Comparative results of ablation experiments on the TripAdvisor dataset.

Method	NDCG@5	NDCG@10	NDCG@15	P@5	P@10	P@15	R@5	R@10	R@15	SR@5	SR@10	SR@15
W/O FE	0.3032	0.2944	0.2857	0.0513	0.0824	0.0993	0.2915	0.3048	0.3139	0.5874	0.5717	0.5581
W/O SSE	0.2976	0.2885	0.2791	0.0498	0.0789	0.0946	0.2860	0.2957	0.3011	0.5439	0.5326	0.5242
**Ours**	0.3202	0.3117	0.3036	0.0589	0.0936	0.1088	0.3045	0.3196	0.3303	0.621	0.6013	0.5897

According to the experimental data in the table, the variant model with some modules removed showed varying degrees of decline in various indicators in the ablation comparison experiments on different datasets. According to Table 4, the W/O FE model performs well in the MovieLens-1M dataset NDCG@15 The decrease from 0.1877 to 0.1698 is 9.5%; P @ 15 decreased from 0.1316 to 0.1143, with a decrease of 13.1%. According to Table 5, in the MovieLens-20M dataset, NDCG@15 decreased from 0.2588 to 0.2337, with a decrease of 9.7%; P @ 15 decreased from 0.1801 to 0.1664, with a decrease of 7.6%. According to Table 6, in the TripAdvisor dataset NDCG@15 The decrease from 0.3036 to 0.2857 is 5.8%; P@15 decreased from 0.1088 to 0.0993, with a decrease of 8.7%. Through experimental data analysis, it can be concluded that removing the feature embedding layer from the original model will have a certain degree of impact on various performance indicators of the model. One possible reason is that the feature embedding layer can grasp the contextual connections between tokens in the input sequence, better evaluate the impact of each token on the deep semantics of other tokens, and thus form a feature embedding that can preserve the original feature distribution more completely and be more robust.

For the W/O SSE model, the decline in various evaluation indicators cannot be ignored, such as in the MovieLens-1M dataset NDCG@15 The decrease from 0.1877 to 0.1646 is 12.3%; P@15 decreased from 0.1316 to 0.1056, with a decrease of 19.7%. In the MovieLens-20M dataset, NDCG@15 decreased from 0.2588 to 0.2281, with a decrease of 11.9%; P@15 decreased from 0.1801 to 0.1579, with a decrease of 12.3%. In the TripAdvisor dataset NDCG@15 The decrease from 0.3036 to 0.2791 is 8.1%; P@15 decreased from 0.1088 to 0.0946, with a decrease of 13.0%. Based on the analysis of the experimental data above, it can be concluded that the weighted scenario scoring formula has the greatest impact on model performance. This may be due to the fact that weighted scenario scoring can finely calculate the compatibility between each item in the candidate list and the user’s interests and preferences. Losing weighted scenario scoring will have a certain impact on the rating of each item in the list for scenarios that are not related to the user’s interests and preferences, resulting in higher ratings for items that should not match the user’s interests and preferences, leading to a decrease in recommendation performance.

## 5. Conclusions

We propose a scene-weighted reranking framework to enhance preference matching in personalized recommendations by addressing local item relevance. It defines “scene” as clusters of semantically related items, establishing them as fundamental reranking units. The model partitions candidate lists into scenes, employs graph neural networks for scene representation learning, and utilizes multi-head attention to generate high-fidelity scene embeddings. To resolve efficiency degradation from item-scene ambiguity, we design a scene-weighted scoring mechanism that computes item scores by: (1) measuring similarities between target items and key items across scenes, and (2) weighting these against scene-user preference matching scores. This dual-perspective approach ensures accurate item positioning in final sequences while comprehensively reflecting user preferences. By integrating advanced neural architectures, our framework captures both local scene relationships and global preference alignment. Experiments show significant improvements over benchmarks across multiple metrics, validating its capability to deliver precise, preference-aware recommendations.
